# Human and rat renal proximal tubule in vitro models for ADME applications

**DOI:** 10.1007/s00204-025-03987-4

**Published:** 2025-03-04

**Authors:** Olivia C. Klatt, Lenya de Brouwer, Femke Hendriks, Eva-Maria Dehne, Beren Ataç Wagegg, Paul Jennings, Anja Wilmes

**Affiliations:** 1 Department of Chemistry and Pharmaceutical Science, Amsterdam Institute of Molecular and Life Sciences, Vrije Universiteit Amsterdam, De Boelelaan 1108, 1081 HZ Amsterdam, The Netherlands; 2 TissUse GmbH, Berlin, Germany; 3 Amsterdam Institute of Molecular and Life Sciences (AIMMS), Vrije Universiteit Amsterdam, Amsterdam, Netherlands

**Keywords:** Renal transport, Renal metabolism, Renal proximal tubular cells, IPSC, Microphysiological system

## Abstract

The kidney is a major organ dictating excretion rates of chemicals and their metabolites from the body and thus renal clearance is frequently a major component of pharmaco-(toxico)-kinetic profiles. Within the nephron, the proximal tubule is the major site for xenobiotic reabsorption from glomerular filtrate and xenobiotic secretion from the blood into the lumen via the expression of multiple inward (lumen to interstitium) and outward transport systems (interstitium to lumen). While there exist several human proximal tubular cell culture options that could be utilized for modelling the proximal tubule component of renal clearance, they do not necessarily represent the full complement of xenobiotic transport processes of their in vivo counterparts. Here, we review available human and rat renal proximal tubule in vitro models, including subcellular fractions, immortalized cell lines, primary cell cultures, induced pluripotent stem cell (iPSC)-derived models and also consider more organotypic cell culture environments such as microporous growth supports, organoids and microfluidic systems. This review focuses on expression levels and function of human and rat renal transporters and phase I and II metabolizing enzymes in these models in order to critically assess their usefulness and to identify potential solutions to overcome identified limitations.

## Plain language summary

The kidney is a major route for xenobiotic excretion and the proximal tubule is a particularly active site for xenobiotic reabsorption, metabolism and secretion. Here, various cell-based kidney models and different methods of maintaining them such as filter inserts and microfluidic systems are reviewed with respect to their xenobiotic transport and metabolism capacity. In addition, we compare rat and human models. This review may be of assistance when choosing a suitable cell-based model for toxicity studies.

## Introduction

Humans are continuously exposed to divergent chemicals (or xenobiotics) from different sources, including industrial chemicals, pesticides, food additives, contaminants of food and water, cosmetics and pharmaceutical agents. Hence, safety evaluations of these substances are vital for regulation and mitigation of toxic compounds. Toxicity studies are carried out in dedicated animal studies that are currently essential to ensure safety for the human population. However, it has long been known that multiple interspecies differences exist, especially in transporters and metabolizing enzymes involved in absorption, distribution, metabolism and excretion (ADME) (Martignoni et al. 2006; Thakur et al. 2024). Such species differences create uncertainties in risk assessment and this, together with ethical concerns, has led to the development of New Approach Methodologies (NAMs) as alternatives to animal methods. The utilization of cell culture systems, specifically of human origin, has been shown to be useful for specific aspects of toxicological evaluations. For example, the combination of human cell systems coupled to omics technologies and/or biokinetic studies where the test chemical is quantified over time have proved useful (Wilmes et al. 2013, 2015; Kramer et al. 2015). In recent years regulatory agencies have been increasing their efforts in integrating NAMs into human health risk assessment. For this purpose, the European Food Safety Authority (EFSA) has developed a roadmap highlighting seven scientific areas that need further development to successfully implement these new approaches (Escher et al. 2022). Within this roadmap the EFSA-funded project ‘ADME4NGRA’ aims to advance toxicokinetic knowledge by utilizing in vitro and in silico models to improve physiological based kinetic (PBK) models and identify potential hazardous chemicals in food and feed.

The kidney is not only one of the key organs involved in ADME processes but it also represents a major target organ of toxicity. Next to its role in excretion, the kidney has several essential physiologic functions, including the regulation of blood pressure, the regulation of erythrocyte numbers via production of the hormone erythropoietin, vitamin D metabolism to produce its active form, acid base balance and maintenance of water and electrolytes in the body. Within the nephron, the proximal tubule is the main site of xenobiotic reabsorption, secretion and metabolism, being equipped with a wide variety of transporters, receptors, channels and metabolizing enzymes (Knights et al. 2013; Chen et al. 2022). Many of these xenobiotic handling processes are of major importance to renal xenobiotic clearance and renal toxicity manifestation. Currently, there are multiple human and rat-based in vitro models available that can be used to study chemical-induced nephrotoxicity. However, the presence or absence of xenobiotic handling transporters and metabolizing enzymes varies greatly between these models and will thus impact ADME parameters including rates of cellular uptake and efflux, bioactivation and accumulation of parent compounds or metabolites within the cell systems. Furthermore, culture conditions can also impact the phenotype of cultured cells or might change the exposure scenario. For example, utilization of microporous filter inserts creates an apical and a basolateral compartment where the cells form a biologic barrier similar to in situ. Also, microfluidic systems provide medium flow within the system, which better emulates the physiological situation.

The aim of this study was to review the literature for information pertaining to gene expression, protein levels and functional capacity of renal transporters and metabolizing enzymes of the available human and rat renal proximal tubular in vitro models, and to outline whether these aspects might be subject to change under different culture conditions.

### Available human and rat in vitro systems

There are several human and rat-derived renal proximal tubule in vitro systems available. The simplest among these models are subcellular fractions that are prepared from homogenized renal tissue. After centrifugation of the tissue with at least 9000*g*, the obtained supernatant is the S9 fraction. A further centrifugation step at 100,000*g* separates the S9 fraction into the microsomal and the cytosolic fraction (Riches et al. 2009; Scotcher et al. 2017). The microsomes contain the endoplasmic reticulum fraction consisting mostly of cytochrome P450 (CYP) and membrane-bound UDP-glucuronosyltransferase (UGT) enzymes. For microsomal incubations both CYPs and UGTs need cofactors to activate the enzymes, which for CYPs is a NADPH-regeneration system, while alamethicin and UDP-glucuronic acid are needed for UGTs (Al-Jahdari et al. 2006; Omura et al. 2021). The cytosolic fractions consist mostly of phase II enzymes such as glutathione-S-transferase (GST) and sulfotransferase (SULT), which require cofactors glutathione (GSH) and 3′-phosphoadenosine-5′-phosphosulfate (PAPS) for activity (Riches et al. 2009; Scotcher et al. 2017; Zhang et al. 2004). Renal subcellular fractions are commercially available for both rats and humans. These fractions are useful for investigating species-specific renal metabolism as has been demonstrated with trichloroethylene (Capinha et al. 2021).

Cell based systems offer more complexity than subcellular fractions. Frequently used human proximal tubular cell lines include HK-2 (human kidney 2) cells, ciPTEC (conditionally immortalized proximal tubular epithelial cells) and RPTEC/TERT1 (tert-transfected renal proximal tubular cells). HK-2 cells were immortalized using the Human Papilloma Virus E6/E7 genes by Ryan et al. (1994). Thus, they have a cancerous phenotype, are not contact-inhibited and are highly glycolytic. ciPTEC were generated from urinary human renal cells by employing a combined immortalization approach using the temperature-sensitive mutant tsA58 of SV40 large T antigen (SV40T) and the human telomerase reverse transcriptase (hTERT) (Wilmer et al. 2010). These cells proliferate when cultured at 33 °C, and display a non-proliferative, more mature phenotype in response to 10 days in culture at 37 °C. ciPTEC have also been utilized to overexpress certain transporters such as ciPTEC-OAT1 and ciPTEC-OAT3 cells, which have overexpression of organic anion transporter (OAT)1 and OAT3, respectively (Nieskens et al. 2016). The RPTEC/TERT1 cell line was generated by overexpression of the catalytic subunit of human telomerase without using oncogene introduction (Wieser et al. 2008). These cells exhibit a normal karyotype, use oxidative phosphorylation as the main energy source in a matured stage and have many of the expected functions and expression profiles of proximal tubule cells (Wieser et al. 2008; Aschauer et al. 2013). RPTEC/TERT1 are fully contact inhibited, display dome formation (indication of vectorial solute and water transport), form a barrier and have a stable transepithelial electrical resistance (TEER) that is associated with expression of proximal tubular specific tight junction proteins, including claudin 2 and 10 (Wilmes et al. 2014; Aschauer et al. 2013). There are also RPTEC/TERT1 cell line derivatives available with overexpression of certain transporters, like OAT1, OAT3 and organic cation transporter (OCT)2 (Tobin et al. 2023). Another human renal proximal tubular cell line, used less frequently, is the RPTEC-SAK7K clone. These cells have been immortalized using a zinc finger nuclease and exhibit a relatively low TEER (Vormann et al. 2018). Available rat renal cell lines include the normal rat kidney (NRK)−52E cells that were isolated as a clone with epithelial morphology from the spontaneously immortalized mixed culture of NRK cells (de Larco and Todaro 1978). While the NRK-52E cell line is extensively used in toxicological evaluations it does not appear to express certain tight junction proteins (Limonciel et al. 2012).

Primary human renal proximal tubular epithelial cells (hPTEC) and primary rat renal proximal tubular epithelial cells (rPTEC) can either be generated from fresh tissue or can be obtained from commercial sources (Zhang et al. 2015; Nieskens et al. 2020; Bajaj et al. 2020; Gledhill et al. 2022; Jennings et al. 2007). Typically, PTEC are obtained by first dissecting the medulla from the cortex, mincing the cortex and performing collagen digestion. Digested cortex contains glomeruli and tubular fragments (approx. 65% proximal), which are separated by sieving. The tubular fragments may be further enriched by density centrifugation. Fragments are then seeded to produce the cell culture. Primary cells have several advantages over cell lines or induced pluripotent stem cell (iPSC)-derived systems as they are typically isolated from normal kidney tissue and thus display an already differentiated phenotype at isolation. Disadvantages of primary cells include their limited availability and their requirement for constant new supply, as they have a very limited number of population doublings before they enter replicative senescence. Also, the source of the tissue is anonymized and there is usually no possibility to select a donor.

A potential disadvantage of human cell lines (and animal studies) is the lack of genetic diversity. iPSC-derived systems offer the possibility to test multiple different donors that are easily available from several biobanks, including the European Biobank for induced pluripotent stem cells (EBiSC). Also, source material is easily obtained from skin biopsies, blood or even fresh urine. Generating iPSC from somatic nucleated cells is relatively straight forward and not very time intensive. Furthermore, undifferentiated iPSCs can be expanded indefinitely and thus do not require constant supply of fresh material. iPSC differentiation into proximal tubular cells is advancing. As an example, Chandrasekaran et al. described a differentiation protocol to differentiate iPSC into renal proximal tubular-like cells (PTL) within 14 days. These PTL can additionally be sub-cultured and cryopreserved once with the addition of the TGF-beta receptor inhibitor GW788388 (Chandrasekaran et al. 2021; Meijer et al. 2024). PTL show a polarized phenotype and express the tight junction associated proteins occludin and ZO3. Furthermore, their function was assessed by cAMP levels in response to parathyroid hormone (Chandrasekaran et al. 2021), which is considered a key discriminator from distal tubular phenotypes. In addition to protocols that derive proximal tubular-like cells directly, several protocols to induce renal organoids derived from iPSC or primary kidney tissue samples have been published (Taguchi et al. 2014; Morizane et al. 2015; Takasato et al. 2016; Przepiorski et al. 2018; Schutgens et al. 2019; Uchimura et al. 2020; Wang et al. 2021). Takasato et al*.* and Morizane et al*.* were among the first to report methods to derive kidney organoids from iPSC and more recently these protocols have been improved to enhance organoid maturity (Morizane et al. 2015; Takasato et al. 2016; Przepiorski et al. 2018; Howden et al. 2019; Vanslambrouck et al. 2022; Yousef Yengej et al. 2023). Furthermore, kidney organoids derived from adult human tissue, referred to as kidney tubuloids, have been reported (Schutgens et al. 2019; Lindoso et al. 2022; Nguyen et al. 2022). Activity developing rat iPSC-derived renal lineages is limited (Kitamura et al. 2015; Ueno et al. 2022).

### Available microphysiological systems

Microfluidic kidney-on-chip models attempt to better replicate the cells’ in vivo environment by the application of flow-mediated fluid shear stress. The perfusion of media may replicate urinary and blood flow and allow to study the effects of physical and biochemical cues on cellular behavior. It was reported that microfluidic conditions lead to a maturation of tissue phenotype as demonstrated by relevant marker expression, barrier integrity and cellular polarization (Jang et al. 2013). Moreover, multi-tissue interactions may be assessed by combining multiple organ equivalents in a common media circuit. Microfluidic kidney-on-chip models, including, hydrogel-based platforms, membrane-bound models and multi-tissue systems will be presented below based on their design principles. Firstly, the use of hydrogel-based platforms allows to model complex tissue architecture and cellular interactions. Here, cylindrical channels that can be coated with extracellular matrix (ECM) proteins can be seeded with kidney glomerular or tubular cells, to which microfluidic perfusion can be introduced via input and output ports. Hydrogel-based platforms are divided into those featuring a single channel for one-sided perfusion (Vriend et al. 2018) and those possessing two adjacent channels allowing a dual-perfused culture setup (Nieskens et al. 2020; Vormann et al. 2021; Aceves et al. 2022). These dual-perfused models enable the integration of additional cell types, such as endothelial cells in a defined spatial arrangement. Here, cellular crosstalk and the effects of interstitial flow on cell behavior may be studied. Hydrogel-based microfluidic platforms were shown to generate leak-tight proximal tubule models with in vivo-relevant marker expression and transporter activity. These models with advanced renal epithelial characteristics were proposed for screening cellular uptake and direct toxic effects of small molecules (Sakolish et al. 2023). Secondly, membrane-bound models divide the culture device into an apical and basolateral compartment by a porous supporting structure, often composed of polycarbonate, polydimethylsiloxane (PDMS) or polyethersulferone (Jang et al. 2013; Maschmeyer et al. 2015; Zhang and Mahler 2021). Both sides of the membrane may be coated by ECM proteins and seeded with epithelial and endothelial cells to form a barrier that can be monitored by permeability dyes or TEER measurements (Ferrell et al. 2010). For the latter, electrodes are incorporated into both compartments enabling online measurements of barrier integrity (Wang et al. 2022). Membrane-bound kidney-on-chips can also be grouped into systems showing single-side perfusion or systems with dual-perfusion mode. Some of these systems also offer compartments that fit commercially available filter inserts leading to models being perfused on one-side (Lin et al. 2020; Nguyen et al. 2023). These systems are especially valuable in multi-tissue co-cultures and will be discussed below. Microfluidic devices that were specifically designed for kidney-on-chip purposes often have two adjacent channels separated by a membrane with a dual-perfusion setup. Perfusion of glomerular or tubular cells by two different flow rates on either side of the membrane was reported to show physiologically relevant levels of cellular polarization and respective marker expression (Jang et al. 2013; Ishahak et al. 2020). These dual-perfused systems are often used for studying consecutive processes like the filtration and reabsorption of substances in combined glomerular-tubular models. Lastly, multi-tissue systems allow for the co-cultivation of different cell types within the same organ, such as podocytes, proximal tubule and endothelial cells on consecutive membranes to model processes relating to the whole nephron unit. It was shown that these co-cultures more closely replicate physiologic albumin filtration and glucose reabsorption compared to mono-cultures (Qu et al. 2018; Zhang and Mahler 2021, 2023). Drug-induced kidney injuries by cisplatin and doxorubicin (aka adriamycin) were successfully modeled and glucose clearance, albumin filtration and lactate dehydrogenase release were compared to healthy controls. Additionally, these systems allow for introduction of different organs on the same chip. Several multi-organ studies using kidney systems have been reported, including kidney and liver (Theobald et al. 2019), kidney and heart (Gabbin et al. 2023) and kidney, liver, intestine and skin (Maschmeyer et al. 2015).

Kidney-on-chip models find their application in various contexts of use ranging from screening toxic effects to modeling disease and evaluation novel therapeutic approaches. The design principle of the underlying microfluidic platform defines the biologic complexity and high-throughput capability of the related assays on-chip. Highly complex multi-tissue systems enable the assessment of systemic interactions, precision medicine approaches and effects of therapeutic interventions, whereas single-tissue systems enable a higher throughput in a physiologically relevant assay format.

### Transport of human and rat renal in vitro models

The proximal tubule exhibits a high activity of sodium potassium ATPase (Na^+^/K^+^-ATPse) which is a multi-protein enzyme of the P-type ATPases. It resides in the basolateral membrane and expels 3 Na^+^ ions from the cytosol in exchange for 2 K^+^ extracellular ions. As such Na^+^ is decreased in the cytoplasm, establishing a sodium gradient for several secondary active symporters and antiporters at the apical membrane. The activity is ATP dependent and much of the mitochondrial activity of the proximal tubule is utilized to fuel this process.

Chemicals enter renal proximal tubular cells via passive diffusion (simple and facilitated diffusion) and active transporters (primary active and secondary active, including symporters and antiporters). Transporters are typically grouped into (1) ATP-binding cassette (ABC) transporters that are located on the apical (aka urinary or luminal) side of the proximal tubulus cells and are involved in active, ATP-driven efflux of compounds and (2) solute carrier family (SLC) located on either apical or basolateral (aka blood) side that are involved in secondary active transport that is linked to transport via gradients, e.g., electrochemical gradients generated by the basolateral expressed Na^+^/K^+^-ATPse. In recent years these renal transporters have been extensively reviewed by Chen et al. (2022) and Łapczuk-Romańska et al. (2023). In short, organic anion transport on the basolateral side is mediated by multi-substrate specific SLC22A6 (OAT1) and SLC22A8 (OAT3) and organic anion-transporting polypeptides SLCO4C1 (OATP4C1) (Vallon et al. 2008). On the urinary side, SLC22A11 (OAT4) is involved in organic anion transport (Chen et al. 2022). Organic cations are taken up on the basolateral side by OCTs of which SLC22A2 (OCT2) is the predominant isoform in the kidney found within the proximal tubule. SLC22A1 (OCT1) was first identified in rat kidneys, but in human kidneys OCT1 is only expressed at relatively low levels compared to other tissues, such as the liver (Karbach et al. 2000). On the apical side organic cation/carnitine transporters novel SLC22A4 (OCTN1) and SLC22A5 (OCTN2) are involved in the bidirectional transport of zwitterions and the multidrug and toxin extrusion transporters SLC47A1 (MATE1) and SLC47A2 (MATE2K) transport these organic cations via antiport of H^+^ into the primary urine (Motohashi and Inui 2013). MATE transporters share a largely overlapping substrate specificity with OCTs. On the apical side, the ABC efflux transporter P-glycoprotein ABCB1 (P-GP; MDR1), multidrug resistance proteins ABCC (MRPs) and the breast cancer resistance protein ABCG2 (BCRP) are located in the brush border membrane. These transporters mediate the unidirectional efflux of a wide range of substrates. The uptake transporters OAT1 and 3 and efflux transporters MRP2 and 4 display a large substrate overlap such as the polyphenol resveratrol (Chen et al. 2022; Jia et al. 2016). In addition, compounds may be taken up from the lumen via receptor-mediated endocytosis, that is orchestrated through proximal tubule low-density lipoprotein receptor-related protein (LRP2/megalin). The megalin/cubilin system is located within the brush border of the apical membrane of the proximal tubulus and is involved in the uptake of xenobiotics such as protein-bound heavy metals and aminoglycosides. Heavy metals bind to metallothionein, which undergoes a megalin-facilitated reuptake in the renal cells (Klassen et al. 2004). These exogenous compounds often accumulate in the kidney and in turn lead to nephrotoxicity, reviewed in detail recently (Chen et al. 2022; Łapczuk-Romańska et al. 2023).

**Fig. 1:**
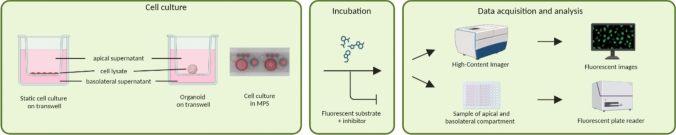
Assessment of functional transport using a fluorescent substrate and an inhibitor

Human renal cell systems vary greatly with regards to reported transporter expression and function, but a major advantage of in vitro models is the possibility to measure functional transport across the apical or basolateral membranes. For these functional studies, cells can be cultured on filter inserts where they should first form a tight monolayer to assess functional transport accurately. Representative TEER values for hPTEC and RPTEC/TERT1 cells range from 80 to 150 ohms*cm^2^ (Wieser et al. 2008; Secker et al. 2019). In contrast, HK-2 cells do not tend to form high TEER values (Wieser et al. 2008) and are therefore often used on plastic only. Once the cells are fully matured on filter inserts and show a tight monolayer, they can be treated with the compound of interest from either apical or basolateral side, in the presence or absence of inhibitors specific to the renal transporter. Compounds can then be quantified via analytical methods, including HPLC and LC–MS in all three compartments: apical and basolateral supernatants and intracellular. Alternatively, the uptake and transport of a fluorescence dye can be measured across the membrane of live cells via fluorescence microscopy, for example with a high-content imager. This can be coupled to measurements of the fluorescent dye in the supernatant of the apical and basolateral medium using a fluorescent plate reader (Fig. 1). In general, these transport studies can also be conducted on a plastic plate, but it should be noted that then only one side of the cells (apical or basolateral) is exposed to the fluorophore, thus making the other side inaccessible (Koepsell et al. 2007; Morrissey et al. 2013). For the determination of OAT and OCT specific transport, several fluorescent dyes and inhibitors have been reported and extensively reviewed (Koepsell et al. 2007; Giacomini et al. 2010; Morrissey et al. 2013). One example that is widely used for OAT transport is the fluorescent dye 6-carboxyfluorescin (6-CF) as a substrate, while probenecid is a commonly used inhibitor (Nieskens et al. 2016; Caetano-Pinto et al. 2016; Meijer et al. 2024). The fluorescence substrate 4-(4-(dimethylamino)styryl)-*N*-methylpyridinium iodide (ASP) is often administered for OCT transport studies and quinidine or cimetidine are widely used as inhibitors of OCTs (Caetano-Pinto et al. 2016; Meijer et al. 2024). For ABC efflux transporters such as P-GP, calcein-AM is a frequently used fluorophore while cyclosporine A can be added to inhibit these transporters (Chen et al. 2022). However, it should be noted that functional activity cannot be assessed easily at an individual isotype level of transporter class, as most transporters overlap largely in substrate and inhibitor specificity. For example, ASP can simultaneously act as a substrate for OCTs and MATEs, 6-CF can simultaneously be transported by OAT1 and 3 (Kido et al. 2011) and calcein AM is a substrate for several ABC transporters. Functional megalin-mediated endocytosis can be investigated with fluorescently labeled albumin as previously reported (Chandrasekaran et al. 2021).

The human renal proximal tubular cell lines mentioned above have been employed in multiple studies to assess transport capacities. The HK-2 cell line has been reported to exhibit limited mRNA expression of renal transporters, including P-GP, MRP1-6 and OATP4C1 compared to expression levels in human renal cortex samples (Jenkinson et al. 2012). Many ABC efflux transporters are functional in HK-2 cells, which is a common characteristic in most cell systems with a cancerous phenotype (Table 1) (Romiti et al. 2002; Jenkinson et al. 2012). ciPTEC have been shown to express relevant transporters including P-GP, BCRP, MRP4 and OCT2 at mRNA and protein level (Wilmer et al. 2010; Schophuizen et al. 2013; Jansen et al. 2014) that have been reported to be functional active (Table 1) (Caetano-Pinto et al. 2016). RPTEC/TERT1 exhibit many proximal tubular drug transport systems on mRNA and protein level, including expression of OAT2, OAT4, OCT2, OCT3, OCTN2, MATE1, MATE2K, P-GP, MRP2, MRP4, MRP5 and BCRP (Wieser et al. 2008; Aschauer et al. 2015; Secker et al. 2018; Saib et al. 2021). Functional transport was reported for OCT, P-GP and BCRP transporter in this model cultured in filter inserts (Table 1) (Chandrasekaran et al. 2021; Meijer et al. 2024). A 3D RPTEC/TERT1 cell model was established with increased mRNA expression for several SLC and ABC transporters. Interestingly, this more complex model showed not only functional OCT but also OAT transport, measured by ASP and lucifer yellow, respectively (Table 1) (Secker et al. 2018). Notably, most of the human cell lines fail to report mRNA, protein or functional megalin (Chandrasekaran et al. 2021; Jenkinson et al. 2012). While albumin reabsorption has been reported in ciPTEC, immunofluorescence staining and western blot failed to show the presence of megalin in this cell line (Wilmer et al. 2010). In addition, OAT1 and OAT3 could not be detected in any of the cell lines when cultured conventionally. Therefore, transfected OAT1 and OAT3 cell lines of HK-2, ciPTEC, and RPTEC/TERT1 have been developed and functional transport has been reported (Nieskens et al. 2016; Sakolish et al. 2023).

**Table 1:** Summary of the expression and functionality of transporters in static human and rat in vitro systems

Test system	Species	Transporter				References
		SLC		ABC	Other	
		Organic cation	Organic anion			
Renal tissue	Human	SLC22A2 (OCT2) P SLC22A3 (OCT3) P (very low) SLC22A4 (OCTN1) P SLC22A5 (OCTN2) P (low) SLC47A1 (MATE1) P SLC47A2 (MATE2K) P (low)	SLC22A6 (OAT1) P SLC22A7 (OAT2) P (low) SLC22A8 (OAT3) P SLC22A11 (OAT4) P (low) SLCO2A1 (OATP2A1) P (low) SLCO4C1 (OATP4C1) P (low)	ABCB1 (P-GP) P ABCC1 (MRP1) P ABCC2 (MRP2) P ABCC3 (MRP3 P ABCC4 (MRP4) P ABCC6 (MRP6) P ABCG2 (BCRP) P (very low)	LRP2 (megalin) mRNA SLC15A1 (PEPT1) mRNA SLC15A2 (PEPT2) mRNA SLC16A1 (MCT1) P	Basit et al. (2019), Thakur et al. (2024) and Mcevoy et al. (2022)
HK-2 cell line	Human		SLCO4C1 (OATP4C1) mRNA/F	ABCB1 (P-GP) mRNA/P/F ABCC1 (MRP1) mRNA/F* ABCC2 (MRP2) mRNA/F* ABCC3 (MRP3) mRNA/F* ABCC4 (MRP4) mRNA/F* ABCC5 (MRP5) mRNA/F* ABCC6 (MRP6) mRNA/F*	SLC16A1 (MCT1) mRNA/F	Romiti et al. (2002), Mutsaers et al. (2011) and Jenkinson et al. (2012)
ciPTEC cell line (wild type)	Human	SLC22A1 (OCT1) mRNA SLC22A2 (OCT2) mRNA/P/F*		ABCB1 (P-GP) mRNA/P/F ABCC2 (MRP2) mRNA ABCC4 (MRP4) mRNA/P/F* ABCG2 (BCRP) mRNA/P/F	LRP2 (megalin) F	Schophuizen et al. (2013), Jansen et al. (2015), Caetano-Pinto et al. (2016) and Wilmer et al. (2010)
RPTEC/TERT1 cell line	Human	SLC22A1 (OCT1) mRNA SLC22A2 (OCT2) mRNA/P/F* SLC22A5 (OCTN2) mRNA/P SLC47A1 (MATE1) mRNA/P/F* SLC47A2 (MATE2K) mRNA/P/F*	SLC22A7 (OAT2) mRNA SLC22A11 (OAT4) mRNA	ABCB1 (P-GP) mRNA/P/F ABCC1 (MRP1) mRNA/P ABCC3 (MRP3) P ABCC4 (MRP4) mRNA/P ABCC5 (MRP5) mRNA/P ABCC6 (MRP6) P ABCG2 (BCRP) mRNA/P/F		Wieser et al. (2008), Aschauer et al. (2015), Saib et al. (2021) and Meijer et al. (2024)
RPTEC/TERT1 (3D sandwich culture, Matrigel)	Human	SLC22A1 (OCT1) mRNA/F* SLC22A4 (OCTN1) mRNA SLC22A5 (OCTN2) mRNA SLC47A1 (MATE1) mRNA SLC47A2 (MATE2K) mRNA	SLC22A7 (OAT2) mRNA/F* SLC22A8 (OAT3) mRNA/F* SLC22A11 (OAT4) mRNA/F*	ABCB1 (P-GP) mRNA/P/F ABCC1 (MRP1) mRNA ABCC2 (MRP2) mRNA ABCC3 (MRP3) mRNA ABCC4 (MRP4) mRNA ABCC5 (MRP5) mRNA ABCG2 (BCRP) mRNA		Secker et al. (2018)
iPSC-derived proximal tubular-like cells (Chandrasekaran)	Human	SLC22A2 (OCT2) mRNA/P/F* SLC22A4 (OCTN1) mRNA SLC22A5 (OCTN2) mRNA		ABCB1 (P-GP) F ABCC1 (MRP1) mRNA ABCC2 (MRP2) mRNA	LRP2 (megalin) mRNA/P/F SLC15A2 (PEPT2) mRNA	Chandrasekaran et al. (2021) and Meijer et al. (2024)
iPSC-derived proximal tubular-like cells (Kandasamy)	Human	SLC22A2 (OCT2) mRNA SLC22A5 (OCTN2) mRNA	SLC22A6 (OAT1) mRNA SLC22A8 (OAT3) mRNA/P	ABCB1 (P-GP) mRNA	SLC15A1 (PEPT1) mRNA/P	Kandasamy et al. (2015)
hPTEC	Human	SLC22A2 (OCT2) mRNA/P/F SLC22A5 (OCTN2) mRNA SLC47A1 (MATE1) mRNA SLC47A2 (MATE2K) mRNA	SLC22A6 (OAT1) mRNA/F* (inconsistent) SLC22A8 (OAT3) mRNA/P/F* (inconsistent) SLC22A11 (OAT4) mRNA SLCO4C1 (OATP4C1) mRNA	ABCB1 (P-GP) mRNA/F ABCC2 (MRP2) mRNA/F* ABCC4 (MRP4) mRNA/F* ABCG2 (BCRP) mRNA/F	LRP2 (megalin) F	Brown et al. (2008), Van der Hauwaert et al. (2014), Bajaj et al. (2020), Sánchez-Romero et al. (2020), Caetano-Pinto et al. (2022) and Meijer et al. (2024)
Renal tissue	Rat	SLC22A1 (OCT1) P SLC22A2 (OCT2) P SLC22A4 (OCTN1) P (low) SLC22A5 (OCTN2) P SLC47A1 (MATE1) P	SLC22A6 (OAT1) P SLC22A7 (OAT2) P (very low) SLC22A8 (OAT3) P SLCO1A1 (OATP1A1) P SLCO1A3 (OATP1B3) P SLCO2A1 (OATP2A1) P (very low) SLCO4C1 (OATP4C1) P	ABCB1 (P-GP) P ABCC1 (MRP1) P ABCC2 (MRP2) P (very llow) ABCC3 (MRP3) P ABCC4 (MRP4) P ABCG2 (BCRP) P	SLC15A1 (PEPT1) P SLC15A2 (PEPT2) P	Thakur et al. (2024) and Basit et al. (2019)
NRK-52E cell line	Rat	SLC22A1 (OCT1) mRNA SLC22A2 (OCT2) mRNA SLC22A5 (OCTN2) mRNA SLC47A1 (MATE1) mRNA		ABCB1 (P-GP) mRNA ABCC4 (MRP4) mRNA ABCG2 (BCRP) mRNA		Khundmiri et al. (2021) and Lechner et al. (2021)
rPTEC	Rat	SLC22A1 (OCT1) mRNA SLC22A2 (OCT2) mRNA SLC22A5 (OCTN2) mRNA SLC47A1 (MATE1) mRNA	SLC22A6 (OAT1) mRNA SLC22A7 (OAT2) mRNA SLC22A8 (OAT3) mRNA SLCo4C1 (OATP4C1) mRNA	ABCB1 (P-GP) mRNA ABCC1 (MRP1) mRNA ABCC2 (MRP2) mRNA ABCC4 (MRP4) mRNA ABCG2 (BCRP) mRNA	LRP2 (megalin) mRNA/P/F SLC15A2 (PEPT2) mRNA	Weiland et al. (2007) and Lechner et al. (2021)

*mRNA* expression has been reported at mRNA level, *P* expression has been reported at protein level, *F* the activity/functionality of the transporter has been measured, F* Functional transport could not be accounted to specific isotype

In human iPSC-derived proximal tubular models, mRNA expression of several SLC and ABC transporters has been observed. Functional activity of P-GP efflux, OCT transport and megalin-mediated endocytosis was reported for an iPSC-derived PTL model (Table 1) (Chandrasekaran et al. 2021; Meijer et al. 2023, 2024). Kandasamy et al*.* reported OAT1 and OAT3 mRNA expression of another iPSC-derived model, but the transporters have not been tested for functionality (Table 1) (Kandasamy et al. 2015). iPSC-derived organoids cultured with methods described by Moriane et al*.* showed functional OCT and P-GP transporters (Table 2) (Rizki-Safitri et al. 2022). In addition, iPSC-derived organoids differentiated through the protocol described by Takasato et al*.* (Takasato et al. 2016), demonstrated mRNA for OCT2. Shankar et al*.* showed mRNA and functional OAT1 and OAT3 at low levels using a modified version of this protocol (Table 2) (Shankar et al. 2021). Vanslambrouk et al*.* also modified the Takasato protocol and observed higher amounts of P-GP, MRP4, OAT1, OCT2, MATE1 and megalin mRNA. Moreover, these organoids displayed functional megalin-mediated endocytosis, which was increased compared to conventional organoids cultures (Table 2) (Howden et al. 2019; Vanslambrouck et al. 2022). Renal organoids offer exciting opportunities in regenerative medicine; however, some challenges still exist at the current state to easily employ them in ADME and toxicity studies. Due to their complexity, it is often more difficult to access the apical and basolateral compartment simultaneously as one of them tends to be on the inside of the organoid. To overcome this issue, Yousef-Yengej et al*.* cultured iPSC-derived organoids until they resembled the first–second trimester of kidney development, and then transferred them to 2D culture conditions on filter inserts. These so called iPSCod tubuloids established a TEER and displayed functional MRP2/3 transport. However, it should be noted that the TEER varied more than 150 ohms*cm^2^ between different batches and mRNA expression of OAT3 was not improved (Yousef Yengej et al. 2023). Other groups have employed adult renal progenitor cells to differentiate them into so called renal tubuloids that show gene expression of several ABC transporters and showed functional MRP2/4 and OAT transport that was assessed using calcein-AM and fluorescein, respectively (Table 2) (Schutgens et al. 2019; Nguyen et al. 2022). Interestingly, when cultured with conditioned medium and extracellular vesicles derived from transfected ciPTEC-OAT1, mRNA expression of MRP3, OAT1 and OAT3 increased more than twofold, threefold and less than twofold, respectively. In addition, OAT1 protein levels and transport activity were improved under these conditions (Table 2) (Lindoso et al. 2022). The advances in human kidney organoids and tubuloids have been recently reviewed in more depth by Dilmen et al. (Dilmen et al. 2024).

**Table 2:** Summary of the expression and functionality of transporters in human and rat kidney organoids and tubuloids

Test system	Species	Transporter				References
		SLC		ABC	Other	
		Organic cation	Organic anion			
iPSC-derived organoid (Morizane)	Human	SLC22A2 (OCT2) mRNA/F*		ABCB1 (P-GP) mRNA/F		Morizane et al. (2015) and Rizki-Safitri et al. (2022)
iPSC-derived organoid (Takasato)	Human	SLC22A2 (OCT2) mRNA	SLC22A6 (OAT1) mRNA/F SLC22A8 (OAT3) mRNA/F			Takasato et al. (2016) and Shankar et al. (2021)
iPSC-derived organoid (Uchimura, Takasato enhanced)	Human				LRP2 (megalin) mRNA CUBN (cubilin) mRNA	Uchimura et al. (2020)
iPSC-derived organoid (Yousef Yengej, Takasato enhanced)	Human		SLC22A8 (OAT3) mNRA			Yousef Yengej et al. (2023)
iPSC-derived organoid (Vanslambrouk, Takasato enhanced)	Human	SLC22A2 (OCT2) mRNA (low) SLC47A1 (MATE1) mRNA/F* SLC47A2 (MATE2K) F*	SLC22A6 (OAT1) mRNA (low)		LRP2 (megalin) mRNA/P/F CUBN (cubilin) mRNA/P?F	Vanslambrouck et al. (2022)
iPSC-derived organoids (Przepiorski)	Human				LRP2 (megalin) mRNA/P	Przepiorski et al. (2018) and Wang et al. (2021)
Kidney tubuloids	Human	SLC22A2 (OCT2) mRNA	SLC22A6 (OAT1) mRNA/F*	ABCB1 (P-GP) mRNA ABCC2 (MRP2) mRNA/F	LRP2 (megalin) mRNA CUBN (cubilin) mRNA	Schutgens et al. (2019) and Nguyen et al. (2022)
Kidney tubuloids (enhanced)	Human		SLC22A6 (OAT1) mRNA/P/F* SLC22A8 (OAT3) mRNA	ABCC2 (MRP2) mRNA (lower) ABCC3 (MRP3) mRNA ABCG2 (BCRP) mRNA (lower)		Lindoso et al. (2022)
iPSCod tubuloids	Human		SLC22A8 (OAT3) mRNA			Yousef Yengej et al. (2023)
Kidney tubuloids	Rat	SLC47A1 (MATE1) mRNA				Ueno et al. (2022)

*mRNA* expression has been reported at mRNA level, *P* expression has been reported at protein level, *F* the activity/functionality of the transporter has been measured, F* Functional transport could not be accounted to specific isotype

hPTEC have also been reported to express several SLC and ABC transporters and megalin-facilitated endocytosis at a functional level (Table 1) (Brown et al. 2008; Bajaj et al. 2020). However, OAT function of primary proximal tubules is not consistent among studies with some suggesting good function (Brown et al. 2008; Bajaj et al. 2020; Sánchez-Romero et al. 2020) and others reporting no functional activity (Caetano-Pinto et al. 2022; Sakolish et al. 2024; Meijer et al. 2024). Isolation and maintenance culture procedures of human primary renal cells may vary between available hPTEC and this may affect OAT function (Nieskens et al. 2020). Lack of reproducibility and availability are major limitations of hPTEC. In addition, large donor differences have been observed in hPTEC and limited doubling time make their lifespan limited (Lock et al. 2006).

In contrast to human renal in vitro models, limited data is available for rat renal in vitro models. The quality of rat NRK-52E and rPTEC seems to be lower compared to human in vitro systems (Weiland et al. 2007; Jennings et al. 2012; Wegner et al. 2014; Khundmiri et al. 2021; Lechner et al. 2021). A study on mRNA expression in NRK-52E revealed low levels of renal transporters OCT1, OCT2, MATE1, MRP4, OCTN2, P-GP, and BCRP1. Overall, mRNA levels of these transporters are below those of rPTEC (Table 1) (Lechner et al. 2021). In addition, functional megalin-mediated endocytosis has been reported in rPTEC (Barta et al. 2023). In kidney organoids derived from rat tissue, gene expression of MATE1 was reported, while no mRNA for OCT1, OCT2 and megalin was detected (Table 2) (Ueno et al. 2022). It should be noted that data on protein levels or functionality of transporters for NRK-52E cells could not be found in the literature.

### Transport of human renal models in MPS

Numerous studies have been published on renal proximal tubular in vitro models in MPS. Here, only studies that reported on the presence and/or function of xenobiotic handling transporters were included. A limited number of studies using renal cell lines have assessed whether transport function could be improved in MPS. A study by Vriend et al*.* compared renal transport of the overexpressing ciPTEC-OAT1 cell line cultured statically and in the Mimetas OrganoPlate^®^. Gene expression levels of OAT1, OCT2, P-GP and MRP2/4 were similar in both conditions (Table 3) (Vriend et al. 2018). Functional transport of P-GP and MRP2/4 was shown; however, no comparison to static conditions was reported (Vriend et al. 2018). Sakolish et al*.* assessed gene expression levels of renal transporters in RPTEC/TERT1 and the transfected cell line RPTEC/TERT1-OAT1, cultured in the PhysioMimix™ organ-o-a-chip (OoC) system (Sakolish et al. 2024) and showed that mRNA levels of P-GP, ABCB10, MRP1-5 and ABCG2 were similar when cultured under flow versus static conditions. In addition, RPTEC/TERT1-OAT1 cells showed expression of OAT1, SLCO1B1 and SLC10A2 at similar levels in static and under medium flow conditions (Table 3). Specioso et al*.* cultured RPTEC-SAK7K cells (MTOX1030, Sigma) in the Vitrofluid micro-physiological system and showed lower mRNA levels of P-GP and MATE1 in chip-cultured cells compared to static culture (Table 3) (Specioso et al. 2022). In addition to renal cell lines, several studies assessed hPTECs in MPS and compared them to static culture conditions. Nieskens et al*.* assessed the gene expression of a subset of transporters in hPTEC cells (Biopredic Inc. RPT101030) that were cultured in Nortis dual-channel chips (Nieskens et al. 2020). They reported that several transporters and receptors showed increased mRNA expression levels in the MPS culture, including MATE1, MATE2K, BCRP and megalin. In addition, OAT1 was only expressed in cells cultured in the chips. In contrast, P-GP mRNA expression was lower in cells cultured in MPS. Similar results were observed by Caetano Pinto et al., who assessed gene expression of transporters in another hPTEC donor (Biopredic Inc. RPT101029) in the Nortis ParVivo© dual-channel chip (Caetano-Pinto et al. 2022). Interestingly, functional transport assays revealed that P-GP mediated efflux of calcein-AM was similar in the chip compared to static, even though mRNA expression of P-GP was lower (Caetano-Pinto et al. 2022). In contrast, Jang et al. reported increased P-GP and megalin function in hPTEC (Biopredic) grown on their own manufactured chips (Jang et al. 2013). Weber et al. cultured hPTEC in the Nortis chips and reported increased functional transport of MRP2/4 and OAT1/3 in MPS compared to static culture (Table 3) (Weber et al. 2016). Another study by Sakolish et al*.* cultured two different donors (referred to as Lonza N-340, Lonza N-405) of hPTECs in the Mimetas OrganoPlate^®^ and showed that MRP 2/4 function was more inhibitable by MK571 in cells cultured in the OrganoPlate^®^ (Sakolish et al. 2023).

**Table 3:** A summary of transporter expression and function of hPTEC cells and immortalized cell lines in MPS compared to statically cultured cells within the same study

Cell type	Chip type	Lower readouts compared to static	Equal readouts compared to static	Higher readouts compared to static	Expressed in chip, not compared to static controls	References
hPTEC	Nortis dual-channel chip	ABCB1 (P-GP) mRNA	CUBN (cubilin) mRNA SLC22A1 (OCT1) mRNA SLC22A2 (OCT2) mRNA SLC22A4 (OCTN1) mRNA SLC22A5 (OCTN2) mRNA SLC31A1 mRNA SLCO4C1 (OATP4C1) mRNA	ABCG2 (BCRP) mRNA LRP2 (megalin) mRNA SLC22A6 (OAT1) mRNA SLC47A1 (MATE1) mRNA SLC47A2 (MATE2K) mRNA		Nieskens et al. (2020)
				ABCC2 (MRP2) F ABCC4 (MRP4) F SLC22A6 (OAT1) F SLC22A8 (OAT3) F		Weber et al. (2016)
		ABCB1 (P-GP), mRNA	ABCB1 (P-GP), F ABCC2 (MRP2) F ABCC4 (MRP4) mRNA, F	SLC47A1 (MATE1) mRNA SLC47A2 (MATE2K) mRNA SLCO4C1 (OATP4C1) mRNA LRP2 (megalin) mRNA CUBN (cubilin) mRNA SLC22A2 (OCT2) mRNA SLC22A6 (OAT1) mRNA		Caetano-Pinto et al. (2022)
	Mimetas OrganoPlate^®^		ABCB1 (P-GP) F	ABCC2 (MRP2) F ABCC4 (MRP4), F	SLCO2B1 (OATP2B1) mRNA SLC22A6 (OAT1) mRNA ABCG2 (BCRP) mRNA ABCC1 (MRP1) mRNA	Sakolish et al. (2023)
	Own design PDMS chips			ABCB1 (P-GP) F CUBN (cubilin) F LRP2 (megalin) F		Jang et al. (2013)
ciPTEC-OAT1	Mimetas OrganoPlate^®^		ABCB1 (P-GP) mRNA ABCC2 (MRP2) mRNA ABCC4 (MRP4) mRNA SLC22A2 (OCT2) mRNA SLC22A6 (OAT1) mRNA		ABCB1 (P-GP) F ABCC2 (MRP2) F ABCC4 (MRP4) F	Vriend et al. (2018)
ciPTEC, ciPTEC-KIF3a^a^	Mimetas OrganoPlate^®^		ABCB1 (P-GP) mRNA ABCC4 (MRP) mRNA CUBN (cubilin) mRNA LRP2 (megalin) mRNA SLC22A2 (OCT2) mRNA SLC47A1 (MATE1) mRNA SLC47A2 (MATE2K) mRNA	ABCB1 (P-GP) F ABCC2 (MRP2) mRNA, F ABCC4 (MRP4) mRNA, F CUBN (cubilin) F LRP2 (megalin) F		Vriend et al. (2020)
RPTEC-SAK7K	Vitrofluid micro-physiologic system	ABCB1 (P-GP) mRNA SLC47A1 (MATE1) mRNA	ABCC2 (MRP2) mRNA CUBN (cubilin) F LRP2 (megalin) F	CUBN (cubilin) mRNA		Specioso et al. (2022)
	Mimetas OrganoPlate^®^				ABCB1 (P-GP) F ABCC2 (MRP2) F ABCC4 (MRP4) F	Vormann et al. (2018)
RPTEC/TERT1	PhysioMimix™ OoC platform		ABCB1 (P-GP) mRNA ABCB10 mRNA ABCC1-5 (MRP1-5) mRNA ABCG2 (BCRP) mRNA			Sakolish et al. (2024)
RPTEC/TERT1-OAT1	PhysioMimix™ OoC platform		ABCB1 (P-GP) mRNA ABCB10 mRNA ABCC1-5 (MRP1-5) mRNA ABCG2 (BCRP) mRNA SLC10A2 mRNA SLC22A6 (OAT1) mRNA SLCO1B1mRNA	SLC22A6 (OAT1) F		Sakolish et al. (2024)

*mRNA* expression has been reported at mRNA level, *F* the activity/functionality of the transporter has been measured

^a^No comparison to static, but normal versus low flow rate

Several studies have assessed renal transporter expression and/or function in renal organoids cultured in different types of MPS (Table 4) (Aceves et al. 2022; Homan et al. 2019; Schutgens et al. 2019). One study cultured iPSC-derived vascularized kidney organoids in 3D-printed chips and showed increased gene expression levels of megalin, P-GP and SLC34A1 when compared to statically cultured organoids (Homan et al. 2019). Another study showed P-GP activity in kidney tubuloids cultured in the Mimetas OrganoPlate® (Schutgens et al. 2019). Two independent studies cultured vascularized spheroids, consisting of rat or human microvascular endothelial cells (Vec technologies), in the Tissue Dynamics system and reported higher mRNA levels of cubilin, megalin, OCT2 and OAT compared to hPTECs cultured in static conditions (Table 4) (Cohen et al. 2021; Ioannidis et al. 2022). In addition to organoid culture, co-culture of renal cells with other cell types has also been reported in MPS. For example, co-cultures of renal and liver cells were performed in the TissUse system, combining RPTEC/TERT1 or kidney tubuloids with liver spheroids (Nguyen et al. 2022; Lin et al. 2020). mRNA levels for different renal influx and efflux transporters were not affected by culture conditions (Table 4) (Nguyen et al. 2022).

**Table 4:** A summary of transporter expression and function in renal organoids and co-cultures in MPS compared to statically cultured cells within the same study

Cell type	Chip type	Lower readouts compared to static	Equal readouts compared to static	Higher readouts compared to static	Expressed in chip, not compared to static controls	References
RPTEC/TERT1 (+ liver)	TissUse 2-Organ chip				ABCB1 (P-GP) mRNA ABCC2 (MRP2) mRNA	Lin et al. (2020)
2D tubuloid (+ liver)	TissUse 2-Organ chip		ABCC2 (MRP2) mRNA CUBN (cubilin) mRNA LRP2 (megalin) mRNA SLC22A2 (OCT2) mRNA SLC22A6 (OAT1) mRNA			Nguyen et al. (2022)
RPTEC/TERT1 (± HGECs)	Own design	ABCC1 (MRP1) mRNA ABCC4 (MRP4) mRNA ABCC6 (MRP6) mRNA SLC15A1 (PEPT1) mRNA SLC29A2 mRNA SLCO4C1 (OATP4C1) mRNA		ABCC3 (MRP3) mRNA SLC22A3 (OCT3) mRNA SLC22A11 (OAT4) mRNA SLC29A1 mRNA SLC47A1 (MATE1) mRNA SLC47A2 (MATE2K) mRNA		Carracedo et al. (2023)
Vascularised spheroids	Tissue dynamics			CUBN (cubilin) mRNA LRP2 (megalin) mRNA SLC22A2 (OCT2) mRNA SLC22A6 (OAT1) mRNA		Cohen et al. (2021) and Ioannidis et al. (2022)
Kidney tubuloids	Mimetas OrganoPlate^®^				ABCB1 (P-GP) F	Schutgens et al. (2019)
OPTECs (organoid-derived PT epithelial cells)	Own design	ABCC4 (MRP4) mRNA SLC22A5 (OCTN2) mRNA SLC47A2 (MATE2K) mRNA		ABCC2 (MRP2) mRNA ABCG2 (BCRP) mRNA LRP2 (megalin) mRNA SLC22A2 (OCT2) mRNA SLC22A4 (OCTN1) mRNA SLC22A6 (OAT1) mRNA, SLC22A8 (OAT3) mRNA, SLC22A11 (OAT4) mRNA SLC47A1 (MATE1) mRNA		Aceves et al. (2022)
iPSC-derived kidney organoids	Own design, 3D printed			ABCB1 (P-GP) mRNA LRP2 (megalin) mRNA SLC34A1 mRNA		Homan et al. (2019)

*mRNA* expression has been reported at mRNA level, *F* the activity/functionality of the transporter has been measured

### Metabolism in human and rat renal models

Many xenobiotics and endogenous substances undergo biotransformation to facilitate their excretion. This process consists of phase I metabolism, which increases the water solubility and can bioactivate the compound, and phase II metabolism, which further increases the water solubility and inactivates the compound. These biotransformation processes of xenobiotics majorly occur within the intestine, liver and kidney (Almazroo et al. 2017) and often convert parent compounds into non-toxic metabolites. However, in some cases, xenobiotic biotransformation may go the opposite way and lead to bioactivation of the parent compound into toxic metabolites (Capinha et al. 2023a). In vitro systems lacking functional metabolism may therefore incorrectly predict the toxicity of compounds that require bioactivation. While the liver is the primary site for phase I metabolism of xenobiotics, the kidney also contains phase I enzymes such as some isotypes of CYPs, aldehyde oxidase (AO) and carboxylesterase (CES) as well as several phase II enzymes such as UGTs, GSTs and SULTs (Van der Hauwaert et al. 2014; Al-Majdoub et al. 2021; Thakur et al. 2024). Phase I enzymes CYP, CES and AO catalyze the oxidation, reduction and hydrolysis of compounds thus adding or exposing a polar group. In the next step of detoxification UGT, GST, SULT enzymes conjugate a hydrophilic group to the exogenous compounds to increase their polarity and thus facilitate the excretion of the parent compound and their metabolites in the urine (Al-Majdoub et al. 2021).

#### Phase I metabolism

CYP enzymes are part of the phase I metabolism to detoxify chemicals. Of the 57 different identified human CYP genes the CYP1, CYP2 and CYP3 families have been determined as the most significant for xenobiotic metabolism (Nebert et al. 2013). Many pesticides such as pyrethroids, organophosphates, and carbamates are metabolized by CYPs while these chemicals often also induce CYP expression (Abass et al. 2012). In general, the liver (and intestine) has the highest activity of CYPs, but several isotypes can also be found in the kidney (Table 5) (Knights et al. 2013, 2016; Bajaj et al. 2018; Fanni et al. 2021; Dhuria et al. 2021; Mcevoy et al. 2022). The expression of CYP2B6 has been consistently reported in human kidneys, while other CYPs such as CYP3A4 and CYP3A5 are inconsistently reported (Lasker et al. 2000; Mcevoy et al. 2022; Van der Hauwaert et al. 2014; Thakur et al. 2024). In a recent proteomics study, CYP3A5 was only present in 3 out of 15 samples tested, underlying how variable the CYP expression is in humans (Thakur et al. 2024). Renal CYP2B6 catalyzes many different reactions in the metabolism of therapeutic drugs, chemotherapeutics, antiretrovirals, anti-inflammatories, anesthetics, and benzodiazepines such as ketamine and propofol (Al-Jahdari et al. 2006). CYP3A5 metabolizes cortisol, statins, tyrosine kinase inhibitors and many more xenobiotics, while members of the CYP4 family are also expressed in the kidney and metabolize substances like omega-6 fatty acid and arachidonic acid (Table 5) (Knights et al. 2013). Other CYPs such as CYP24A1 and CYP27B1 are involved in the regulation of vitamin D. While CYP27B1 catalyzes the reaction of the inactive vitamin D form calcifediol to the active form calcitriol, CYP24A1 initiates the degradation of this metabolite by hydroxylating the side chain. These two CYPs are predominantly found in the kidney, which highlights the importance of renal CYP metabolism for vitamin D (Bajaj et al. 2018).

**Table 5:** Renal phase I metabolizing enzymes in vivo and in different in vitro models under static culture conditions

Test system	Species	Phase I enzyme						References
		CYP1	CYP2	CYP3	CYP4	Other CYPs	Non-CYPs	
Renal tissue	Human	CYP1A1 mRNA CYP1B1 mRNA	CYP2B6 mRNA/P CYP2C8 mRNA (inconsistent) CYP2C9 mRNA (inconsistent) CYP2C19 mRNA	CYP3A4 mRNA (inconsistent) CYP3A5 mRNA/P	CYP4A11 mRNA/P CYP4F2 mRNA/P CYP4F3 P CYP4F8 mRNA CYP4F11 mRNA CYP4F12 mRNA	CYP24A1 mRNA CYP27B1 mRNA	AO1 mRNA CES1 mRNA/P (low) CES2 mRNA/P	Van der Hauwaert et al. (2014), Basit et al. (2020), Al‐Majdoub et al. (2021), Mcevoy et al. (2022) and Thakur et al. (2024)
Subcellular fractions (S9, microsomes, cytosol)	Human	CYP1A1 F	CYP2B6 F	CYP3A F CYP3A4 P	CYP4A11 P CYP4F2 P/F		AO F CES2 F	Nishimuta et al. (2014), Stiborová et al. (2005), Lash et al. (2008), Knights et al. (2013), Snider et al. (2007), Kozminski et al. (2021) and Nakamura et al. (2016)
HK-2 cell line	Human	CYP1A1 mRNA CYP1B1 mRNA	CYP2B6 mRNA (very low) CYP2D6 mRNA (inconsistent) CYP2J2 mRNA	CYP3A4 mRNA (inconsistent) CYP3A5 mRNA (inconsistent)	CYP4A11 mRNA (inconsistent) CYP4F2 mRNA (very low) CYP4F8 mRNA (very low) CYP4F11 mRNA			Ryan et al. (1994), Van der Hauwaert et al. (2014) and Shah et al. (2017)
ciPTEC cell line	Human	CYP1A1 mRNA CYP1B1 mRNA	CYP2B6 mRNA CYP2C8 mRNA, CYP2C9 mRNA CYP2C19 mRNA CYP2D6 mRNA	CYP3A4 mRNA CYP3A5 mRNA	CYP4A11 mRNA (very low) CYP4F11 mRNA CYP4F12 mRNA			Mutsaers et al. (2013), Caetano-Pinto et al. (2016) and Nieskens et al. (2016)
RPTEC/TERT1 cell line	Human	CYP1A1 mRNA/F (very low) CYP1B1 mRNA/F	CYP2B6 mRNA CYP2D6 mRNA (very low) CYP2J2 mRNA	CYP3A4 mRNA (very low) CYP3A5 mRNA (low)	CYP4A11 mRNA CYP4F2 mRNA (low) CYP4F11 mRNA			Simon et al. (2014), Shah et al. (2017), Capinha et al. (2023a, b)
iPSC-derived PTL (Chandra-sekaran)	Human	CYP1B1 mRNA				CYP20A1 mRNA CYP24A1 mRNA CYP26B1 mRNA		Chandrasekaran et al. (2021)
iPSC-derived PTL (Kandasamy)	Human					CYP27B1 mRNA		Kandasamy et al. (2015)
hPTEC	Human	CYP1A1 mRNA CYP1B1 mRNA	CYP2A6 mRNA CYP2B6 mRNA CYP2C8 mRNA CYP2E1 mRNA	CYP3A4 mRNA/F CYP3A5 mRNA/F	CYP4A11 mRNA		CES2 mRNA CES3 mRNA	Lash et al. (2008), Mutsaers et al. (2013) and Van der Hauwaert et al. (2014)
Renal tissue	Rat	CYP1A1 mRNA	CYP2B1 mRNA CYP2C2 mRNA CYP2C9 mRNA (inconsistent) CYP2C11 mRNA/P CYP2C11 mRNA CYP2C23 mRNA/P CYP2D1 P CYP2E1 mRNA/P	CYP3A1 mRNA CYP3A2 mRNA CYP3A P	CYP4A1 P CYP4A3 mRNA CYP4A8 mRNA CYP4B1 P CYP4F1 mRNA CYP4F2 P CYP4F4 mRNA CYP4F6 mRNA		CES1 mRNA/P CES2 P	Limbutara et al. (2020), Thakur et al. (2024), Weiland et al. (2007) and Gerges and El-Kadi (2023)
Subcellular fractions (S9, microsomes, cytosol)	Rat	CYP1A1 P/F	CYP2A1 F CYP2B P/F CYP2C F CYP2D1 P CYP2E1 P/F	CYP3A F	CYP4A8 P		CES1 F	Schaaf et al. (2001), Nishimuta et al. (2014), Stiborová et al. (2014), Ronis et al. (1998) and Sohlenius-Sternbeck and Orzechowski (2004)
NRK-52E	Rat	CYP1A1 mRNA/F*						Lash et al. (2002), Lechner et al. (2021) and Komatsu et al. (2023)
rPTEC	Rat	CYP1A1 mRNA/F* CYP1A2 F	CYP2C mRNA/F* CYP2C F CYP2D1 F	CYP3A mRNA/F*	CYP4A3 mRNA			Schaaf et al. (2001), Lechner et al. (2021) and Weiland et al. (2007)

mRNA expression reported at mRNA level, *P* expression reported at protein level, *F* the functionality/activity of the enzyme has been measured, F* most of the functionality/activity was lost within first 24 h

**Fig. 2:**
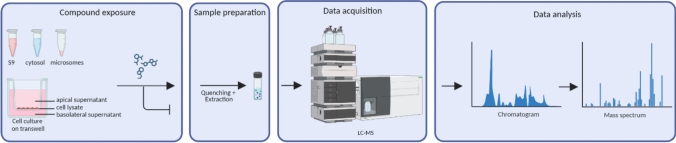
Workflow of the assessment of the metabolic enzyme activity using LC–MS

To assess functional isotype specific metabolic enzyme activity, the in vitro system is exposed to a known substrate of this metabolic enzyme in the presence or absence of a specific inhibitor. The amount of parent compound and the formed metabolites can then be quantified using LC–MS (Fig. 2). While some substrates are quite specific for one CYP isozyme, many CYPs are promiscuous with overlapping substrates, for example ketoconazole which has shown to inhibit CYP1A1 and CYP3A4 (Martignoni et al. 2006). Examples for relatively specific substrates include acetaminophen (CYP1A2), hydroxy-bupropion (CYP2B6), *n*-desethyl-amodiaquine (CYP2C8), 4′-hydroxy-diclofenac (CYP2C9), 4′-hydroxy-mephenytoin (CYP2C19), dextrorphan (CYP2D6) and 1′-hydroxy-midazolam (CYP3A4/5). Well-known inhibitors of these CYPs are furafylline (CYP1A2), thiotepa (CYP2B6), quercetin (CYP2C8), sulfaphenazol (CYP2C9), ticlopidine (CYP2C19), quinidine (CYP2D6) and azamulin (CYP3A4/5) (Liu et al. 2015; Chanteux et al. 2020). In addition to the analysis by LC–MS, luminescent assays such as P450-Glo™ can be used to assess CYP functionality. These assays use the ability of a specific CYP enzyme to metabolize derivates of luciferin. The CYP-formed luciferin reacts with luciferase and the emitted light can be quantified using a luminescent plate reader (Cali et al. 2006). Another way to measure CYP activity, such as CYP1A, is the ethoxyresorufin-O-deethylase (EROD) assay. This assay measures the formation of the fluorophore resorufin via deethylation of the assay substate 7-ethoxyresorufin (Lash et al. 2008).

Human and rat renal in vitro systems for phase I metabolism are shown in Table 5. CYP metabolism in subcellular fractions needs the addition of an NADPH-regeneration system to transport cofactors across the endoplasmic reticulum. These subcellular fractions can be used to measure the activity or intrinsic clearance of substrates by their metabolizing enzyme in milligrams of microsomal protein and can be later scaled according to the microsomal protein present per gram kidney (Scotcher et al. 2016). Of all CYP enzymes in subcellular fractions, the protein levels of CYP4A11 were the highest in human kidney microsomes (HKM) which was similar to the enzyme level reported in liver microsomes (Nakamura et al. 2016). Most CYP enzymes were expressed and functional in human subcellular fractions, making them a well-suited and simple starting model when investigating the metabolism of compounds (Table 5).

mRNA expression of various CYPs has been detected in human renal cell lines, but protein levels for some of these enzymes were only reported in HK-2 and RPTEC/TERT1 cells (Table 5). It should be noted that the variety of CYPs in RPTEC/TERT1 cells is higher in contrast to HK-2. In addition, gene expression of CYP3A4 and CYP3A5 in HK-2 cells has been reported inconsistently and at very low levels (Table 5) (Van der Hauwaert et al. 2014; Shah et al. 2017). In RPTEC/TERT1 cells mRNA expression of CYP1A1 and CYP1B1 was inducible upon treatment with benzo[a]pyrene, with CYP1B1 also being present at low levels prior to the treatment. In addition, the same study reported functional CYP1A/B activity using the EROD assay (Simon et al. 2014). In iPSC-derived PTL the gene expression of various CYPs has been reported (Table 5) and functional analysis is still under investigation. CYP mRNA and enzyme expression in hPTEC varied between tissue samples of different donors, with CYP3A4 mRNA present in all donors and protein expression found in 12 of 13 hPTEC of different donors (Lash et al. 2008; Van der Hauwaert et al. 2014). hPTEC seem to underperform when looking at the CYP activity, since out of CYP1A1, 1A2, 2B6, 2C9, 2C19, 2D6 and 3A4 only activity for CYP3A4 was detectable (Table 5) (Lash et al. 2008). It is of importance that quality controls between different isolation batches are carefully examined. Compared to human systems, rat in vitro systems, such as rat kidney microsomes (RKM), express a higher amount of functional CYP enzymes (Table 5) (Cummings et al. 1999). While CYP activity in cultured rPTEC was initially reported, enzymatic activity of CYP1A1 and CYP3A4 was found to decrease drastically within the first 24 h of culture. CYP2C1 and CYP2D1 activity remained longer, but nearly no activity was detected after 7 days of culture (Table 5) (Schaaf et al. 2001). Interestingly, CYP levels can also be higher in cultured cells compared to biopsy tissues. A protein quantification study of liver samples showed increased CYP2B6 and CYP3A4 protein levels in primary hepatocytes in cell culture conditions compared to human or rat biopsy tissue (Hammer et al. 2021).

Other phase I enzymes that are accounting for metabolism of approximately 30% of all drugs, include AO and CES (Cerny 2016). They can be found in renal in vitro models such as S9 fractions, HK-2 and hPTEC (Table 5) (Van der Hauwaert et al. 2014). AO is present in the cytosol and catalyzes the oxidation of aldehydes to carboxylic acid. Current in vitro hepatic subcellular fractions underestimate the AO elimination of xenobiotics raising the need to include in vitro models of other organs (Nishimuta et al. 2014; Basit et al. 2020; Dhuria et al. 2021; Kozminski et al. 2021). Of all extrahepatic tissue, AO activity was the highest in human renal S9 fractions, but different studies showed varying amounts of AO expression (Basit et al. 2020; Kozminski et al. 2021). In comparison to humans, rats do not seem to express AO on a protein level, underlying yet another interspecies difference in xenobiotic metabolism (Thakur et al. 2024).

#### Phase II metabolism

The phase II enzymes facilitate the excretion of detoxified compounds by adding a polar group to the chemical. UGTs facilitate the attachment of the polar glucuronic acid to a nucleophilic group on the substrate, typically hydroxyl, carboxyl, or amino groups (Miners et al. 2021). Cytosolic SULTs add a sulfuryl group from PAPS to a hydroxyl or amino group of the side chain, thus increasing the detoxification and elimination of drugs (Tian et al. 2015). GSTs conjugate reduced glutathione to hydrophobic and electrophilic exogenous and endogenous substrates to facilitate the elimination of the xenobiotic. The conjugation of GSH can also occur spontaneously inside each cell since they contain 1–10 mM free GSH (Scotcher et al. 2017; Cui et al. 2023). Functional activity of UGTs, GSTs and SULTs can be assessed similar to CYPs using specific substrates and inhibitors and subsequent analysis of the parent compound and its metabolites using LC–MS. The total amount of UGT enzymes is the highest in the liver, nevertheless UGT1A6, 1A9 and 2B7 are most abundant in the human kidney (Table 6) (Ahire et al. 2023).

**Table 6:** Renal phase II and brush border enzyme expression and functionality in vivo and in different in vitro models in static culture conditions

Test system	Species	Phase II enzyme			Brush border	References
		UGT	SULT	GST	GGT	
Renal tissue	Human	UGT1A1 mRNA/P UGT1A3 P UGT1A4 P UGT1A5 mRNA UGT1A6 mRNA/P UGT1A7 mRNA/P UGT1A9 mRNA/P UGT2B4 mRNA UGT2B7 mRNA/P UGT2B10 mRNA UGT2B14 mRNA UGT2B15 P UGT2B17 mRNA	SULT1A1 P SULT1A3 P SULT1B1 P (inconsistent) SULT1E1 P SULT2B1 P	GSTA1 mRNA GSTK1 mRNA GSTM mRNA GSTP mRNA GSTT mRNA (inconsistent)	GGT1 mRNA GGT2 mRNA (very low) GGT5 mRNA (very low) GGT6 mRNA	Van der Hauwaert et al. (2014), Basit et al. (2020), Al‐Majdoub et al. (2021), Mcevoy et al. (2022), Thakur et al. (2024) and Riches et al. (2009)
Subcellular fractions (S9, microsomes, cytosol)	Human	UGT1A6 P/F UGT1A4 P UGT1A7 P UGT1A8 P UGT1A9 P/F UGT2B7 P/F	SULTs F (very low) SULT1A1 P SULT1A3 P SULT1B1 P SULT1E1 P SULT2B1 P	GSTs F		Al-Jahdari et al. (2006), Knights et al. (2016), Scotcher et al. (2017), Omura et al. (2021), Riches et al. (2009), Nakamura et al. (2016) and Harbourt et al. (2012)
HK-2 cell line	Human	UGT1A9 mRNA	SULT1E1 mRNA (very low) SULT2A1 mRNA (low)	GSTA1 mRNA GSTA2 mRNA (low) GSTK1 mRNA GSTP1 mRNA GSTT mRNA	GGTs mRNA	Ryan et al. (1994), Van der Hauwaert et al. (2014) and Shah et al. (2017)
ciPTEC cell line	Human	UGT1A1 mRNA/P UGT1A9 mRNA/P UGT2B7 mRNA/P UGT2B8 mRNA/P	SULT1A1 mRNA SULT1A3 mRNA SULT1E1 mRNA SULT2B1 mRNA	GSTA4 mRNA GSTO mRNA GSTP mRNA		Mutsaers et al. (2013), Nieskens et al. (2016) and Caetano-Pinto et al. (2016)
RPTEC/TERT1 cell line	Human	UGT1A8 mRNA UGT1A9 mRNA UGT2B7 mRNA UGT2B8 mRNA	SULT1E1 mRNA (low) SULT2A1 mRNA (low)	GSTA4 mRNA/P GSTF mRNA GSTK1 mRNA GSTT mRNA (inconsistent)	GGTs F GGT1 mRNA GGT5 mRNA	Simon et al. (2014), Shah et al. (2017) and Capinha et al. (2023a, b)
iPSC-derived PTL (Chandrasekaran)	Human			GSTA4 mRNA GSTK1 mRNA GSTP1 mRNA		Chandrasekaran et al. (2021)
iPSC-derived PTL (Kandasamy)	Human				GGTs mRNA	Kandasamy et al. (2015)
iPSC-derived organoid	Human	UGT2B7 mRNA UGT3A1 mRNA	SULT1A3 mRNA		GGT1 mRNA/F	Wu et al. (2018), Howden et al. (2019), Phipson et al. (2019), Uchimura et al. (2020) and Vanslambrouck et al. (2022)
hPTEC	Human	UGT1A1 mRNA UGT1A6 mRNA UGT2B7 mRNA	SULT1E1 P SULT1E P SULT2A1 P	GSTA4 P GSTP P GSTT P	GGTs mRNA/F	Lash et al. (2008), Mutsaers et al. (2013), Van der Hauwaert et al. (2014) and Sánchez-Romero et al. (2020)
Renal tissue	Rat	UGT1A1 mRNA/P UGT1A2 mRNA (very low) UGT1A3 mRNA UGT1A5 mRNA UGT1A6 P UGT1A7 mRNA/P (low) UGT1A8 mRNA UGT2B1 P UGT2B2 mRNA UGT2B12 mRNA UGT2B15 P	SULT 1A1 mRNA/P SULT1B1 mRNA SULT1C2a P SULT2A1 mRNA SULT2B1 SULT1C2 mRNA	GSTA2 mRNA GSTA4 mRNA GSTM5 mRNA GSTT1 mRNA	GGTs mRNA	Weiland et al. (2007) and Thakur et al. (2024)
Subcellular fractions (S9, microsomes, cytosol)	Rat	UGTs F	SULTs F	GSTs F		Schaaf et al. (2001), Stiborová et al. (2014), Sohlenius-Sternbeck and Orzechowski (2004) and Brunelle and Verbeeck (1996)
NRK-52E	Rat			GSTs mRNA/F	GGT1 mRNA/F	Lash et al. (2002), Lechner et al. (2021) and Komatsu et al. (2023)
rPTEC	Rat	UGTs F	SULTs F SULT1A1 mRNA SULT1C2 mRNA	GSTs F GSTA2 mRNA GSTA4 mRNA GSTM4 mRNA GSTO1 mRNA GSTT1 mRNA	GGTs F GGT1 mRNA GGTP mRNA	Schaaf et al. (2001), Weiland et al. (2007) and Lechner et al. (2021)

*mRNA* expression reported at mRNA level, *P* expression reported at protein level, *F* the functionality/activity of the enzyme has been measured

In vitro studies in human liver microsomes (HLM) and HKM have shown a 1.8- and 2.2-fold higher expression of UGT1A6 and UGT1A9 in the kidney compared to the liver (Knights et al. 2016). However, another study found similar but slightly higher values of UGT1A9 in liver S9 fractions compared to the kidney (Ahire et al. 2023). This difference could be donor-specific. In addition, the location of kidney tissue used for the preparation of subcellular fractions also has an impact on the activity, since intrinsic clearance by UGT2B7 has been found to be 4.5-fold higher in renal cortical microsomes than kidney medullary microsomes (Paraskevi et al. 2007). Similar to CYPs, UGTs in microsomes need alamethicin, a pore-forming peptide, to expose the membrane-bound enzyme and UDP-glucuronic acid to activate it. Glucuronidation clearance of UGT1A9 substrates such as propofol and edaravone was higher in HKM compared to liver microsomes (Soars et al. 2002; Ma et al. 2012). An overall drawback of HKM (and microsomes in general) is that they underestimate in vivo clearance (Al-Jahdari et al. 2006). One possible explanation for this observed effect could be the location of UGTs in the membrane of the endoplasmic reticulum. The methods to expose these UGT enzymes could affect the activity. Another explanation for the underestimation could be an inhibition of the UGTs by fatty acids in microsomes. Moreover, it has been found that the addition of bovine serum albumin to HKM increased the rate of conjugation which is known as the “albumin effect” thus improving the in vitro clearance predictions. However, the impact of this effect on the prediction was drug dependent (Gill et al. 2012).

In iPSC-derived organoids gene expression of UGT2B7 was detected (Wu et al. 2018) and with the additional factors of vasopressin and aldosterone in the late stages of kidney organoid maturation mRNA of UGT3A1 was reported (Uchimura et al. 2020). In hPTEC the enzyme expression of UGT1A1, 1A6 and 2B7 was detected, but the expression levels varied drastically between donors, with UGT1A6 from one donor only being detected day 0, while cells of the other donor showed expression throughout 5 days of culture (Table 6) (Lash et al. 2008). rPTEC showed relatively stable UGT activity over 7 days in culture (Table 6) (Schaaf et al. 2001). The main SULTs involved in xenobiotic phase II metabolism are SULT1A1, SULT1A3 and SULT1B1, which could be detected in cytosolic fractions of kidneys (Table 6) (Riches et al. 2009). SULT1A1 represented 40% of the total SULTs in kidney cytosolic samples. It should be noted that the expression levels displayed large variability between donors. In addition, sex specific differences in SULT activity in humans have been reported, as SULT activity for melatonin metabolites in pooled cytosolic fractions of human kidneys seemed to be higher in females (Tian et al. 2015; Riches et al. 2009). However, when compared to cytosolic fractions of other organs, such as the liver, small intestine and lung, the sulfation rate in the kidney seems to be much lower (Tian et al. 2015; Omura et al. 2021). iPSC-derived organoids reported low amounts of SULT1E1 mRNA expression when matured to whole organoids on day 18 and 25, while SULT1E1 was absent in unmatured organoids (Phipson et al. 2019). In hPTEC enzyme expression of SULT1A3, SULT1E and SULT2A1 were detected with expression levels decreasing for SULT2A1 after 5 days in culture (Table 6) (Lash et al. 2008). In rPTEC SULT enzyme activity was detected within the first 24 h of culturing rPTEC, but not in cells cultured for longer periods (Table 6) (Schaaf et al. 2001).

In human kidneys, mRNA of GSTA1, GSTK1, GSTP1 was present, but GSTT1 expression was only detected in five out of eight samples (Table 6) (Van der Hauwaert et al. 2014). Human and rat kidney microsomes and cytosolic fractions showed activity of the phase II GST enzymes (Table 6). One study comparing the GST activity in HKM and human cytosolic fractions, found that on average 14.5% of total GST activity was found in HKM and 31% of GST activity in cytosolic fractions (Scotcher et al. 2017). In hPTEC enzyme levels of GSTA, GSTT and GSTP were detected. GSTP levels were the lowest, whereas GSTA was the only enzyme decreasing with time the cells were in culture (Table 6) (Lash et al. 2008). In rPTEC, GST activity did not decrease drastically with prolonged culturing time (Table 6) (Schaaf et al. 2001). Immortalized cell lines, iPSC-derived PTL and kidney organoids are far less characterized for renal metabolism, especially for phase II metabolism. Hence, the lack of described phase II enzymes in the renal cell systems may also be due to limited studies investigating the presence of these enzymes. In HK-2 cells several isotypes of UGT, GST and SULT have been reported, while in ciPTEC mainly UGT isotypes have been described. mRNA expression of phase II enzymes GST, UGT, and SULT were found in RPTEC/TERT1 but not for all enzymes protein levels and functional activity have been reported (Table 6) (Simon et al. 2014; Shah et al. 2017; Chandrasekaran et al. 2021). For the rat NRK-52E cell line, we could only find reports on phase II enzyme GST mRNA and function (Table 6) (Lash et al. 2002).

#### Brush border enzymes

The apical brush border membrane of the proximal tubule contains several specific metabolizing enzymes that have been reported to be involved in xenobiotic metabolism. These include aminopeptidases, alkaline phosphatase, neutral endopeptidase, epoxide hydrolase and γ-glutamyl transpeptidase (GGT) which facilitate the reabsorption of compounds by dephosphorylation, hydrolysis, esterification, and proteolysis and act downstream from the phase II enzymes. In this review, we only focused on GGT since these enzymes are essential in the redox-recycling mercapturic acid pathway. In this pathway, GGT facilitates the reuptake of GSH-conjugates into the proximal tubule by cleaving a γ-glutamyl group and thus forming a cysteine S-conjugates. These metabolites are often nephrotoxic (Capinha et al. 2023a).

To measure the GGT activity one can conduct similar experiments to phase I or phase II enzymes. The GSH-conjugate of the parent compound should be administered in the absence and presence of a selective inhibitor such as acivicin. Subsequent LC–MS analysis of the in vitro system detects the parent compound and its metabolites (Weber et al. 2016). In addition, GGT activity can be assessed using a GGT activity colorimetric assay in which the chromogen p-nitroanilide is measured at 418 nm. If active GGT is present, the cleavage of a γ-glutamyl group from L-γ-Glutamyl-p-nitroanilide releases the chromogen (Del Corso et al. 2006). mRNA of GGT was reported in HK-2 cells, RPTEC/TERT1 cells, both iPSC-derived PTL-like cells, iPSC-derived kidney organoids and primary hPTEC (Table 6) (Aschauer et al. 2015; Kandasamy et al. 2015; Shah et al. 2017; Howden et al. 2019; Mihevc et al. 2020; Chandrasekaran et al. 2021; Vanslambrouck et al. 2022). Capinha and colleagues showed that RPTEC/TERT1 cells are a suitable model to predict the bioactivation of glutathione conjugated metabolites of trichloroethylene (i.e., dichlorovinyl, DCV) (Capinha et al. 2023a). In NRK-52E cells, mRNA of GGT was detected but compared to freshly isolated rPTEC the GGT1 levels were much lower (Lechner et al. 2021). Enzyme activity in rPTEC decreased over 7 days in culture (Table 6) (Schaaf et al. 2001).

### Metabolism of renal cell models in MPS

Renal metabolism in proximal tubular cells cultured in MPS has not yet been studied extensively. Sakolish et al. reported mRNA levels of CYP1B1, CYP2B6, CYP3A5 and GGT1 in two different hPTEC donors (Lonza N-340 and Lonza N-405) cultured in the Mimetas OrganoPlate^®^. A comparison with statically cultured cells was not included (Sakolish et al. 2023). Another study by Sakolish et al. reported similar gene expression levels of CYP1B1 and CYP2B6 in RPTEC/TERT1 and RPTEC/TERT1-OAT1 cells under static and MPS conditions. CYP3A5 was not expressed in RPTEC/TERT1 cells, and only present at very low levels in RPTEC/TERT1-OAT1 cells in both static culture and MPS (Sakolish et al. 2024). Weber et al*.* reported functional GGT in hPTEC cultured in the Nortis dual-channel chip (Weber et al. 2016).

### Species differences between rat and human

Overall, multiple species differences in xenobiotic handling metabolizing enzymes and transporters have been reported (Martignoni et al. 2006). Alterations in drug disposition may influence intracellular drug levels, contributing to nephrotoxicity. Therefore, understanding cross-species differences is essential to improve the extrapolation of animal-derived disposition data to humans for risk assessment. Proteomic studies on human and rat tissue showed significant upregulation of several important renal transporters in rats compared to humans (Basit et al. 2019; Thakur et al. 2024). OAT1, OCT2 and OCTN2 expression levels were 2.9-fold, 1.5-fold and 7.4-fold higher in rats, respectively. Similarly, MRP1, MRP3 and MRP4 were 2.9-fold, 5.4-fold and 1.9-fold higher in rats compared to humans, respectively. Moreover, OCT1 expression was quantified in rats, whereas in humans it was under the limit of detection, while the peptide was conserved. In contrast, OAT2, OAT3, OAT4, OATP4C1, OCT3, OCTN1, MATE1 and MRP2 could only be detected in humans and were below the level of quantification in rats (Basit et al. 2019). However, while the trend in multiple proteomics studies is similar, the exact difference in abundance levels varied greatly between studies, for example Basit et al. and Thakar et al. reported 2.9-fold and sevenfold higher levels of OAT1 in rats, respectively. Possible explanations for the discrepancy could be differences in sample size, different sample preparation methods and limits of quantification (Basit et al. 2019; Thakur et al. 2024). Differences in CYP metabolism between human and rat have also been reported and concerns have been expressed on whether we can easily extrapolate from one species to another (Martignoni et al. 2006; Thakur et al. 2024). Proteomics studies have shown that the most abundant CYP form in rat kidneys is CYP2C23, while in human kidneys it is CYP4A11 (Gerges and El-Kadi 2023; Thakur et al. 2024). While some orthologs of CYP are similar in humans and rats, like CYP1A1, others correspond to different CYPs (Hammer et al. 2021). For example, human CYP4A11 corresponds to rat CYP4A1 and human CYP3A5 to rat CYP3A1, CYP3A9 and CYP3A18, respectively. Direct comparison of CYP orthologs between both species have been published by Hammer et al. (2021), Gerges and El-Kadi (2023) and Thakur et al. (2024). For enzymes involved in phase II metabolism, sequences were more comparable with UGT1A6 and UGT1A9 showing 80% and 78% similarity between rats and humans. However, protein abundance differed for some proteins. SULT1A1 was 1.5-fold higher expressed in humans than rats, while SULT1C2A was only present in rats (Thakur et al. 2024).

Interestingly, the effect of sex differences on metabolic enzyme and transporter protein abundance has also been reported in human and rats, with overall 1046 proteins significantly differing between sexes in rats, while only 47 proteins differed in humans (Thakur et al. 2024). In rats, protein levels for CYP2E1 and CYP3A were 1.7-fold and 2.4-fold higher in female rats. CYP1A1 was only detected in female animals (Gerges and El-Kadi 2023). In contrast, SULT1A1 was 1.5-fold higher in male rats. In humans, AO protein abundance was twofold higher in males (Thakur et al. 2024). Protein abundance for renal transporter on the other hand differed significantly in rats with no sex differences reported in humans (Basit et al. 2019; Thakur et al. 2024). OAT1, OCT2 and OCTN2 were 1.3-fold, 1.4-fold and 1.4-fold higher in male compared to female rats. Similar results were reported for P-GP and BCRP that were both 1.6-fold higher in male rats. In contrast, MRP3 protein abundance was slightly higher (1.1-fold) in female rats (Basit et al. 2019).

Large discrepancies have been reported for human drug clearance when compared to the rat clearance data, often resulting in overestimation of drug clearance in humans (Jansen et al. 2020). Improving our understanding of species differences using rat renal in vitro systems would be a logical step; however, the current rat cell systems may not be best fit due to significant quality differences between rat and human in vitro systems. Especially, the only available rat cell line NRK-52 K cells might not be a suitable model to predict risk assessment of xenobiotics as it displays a cancerous phenotype, and it was found in a proteomics study comparing whole-rat kidney to NRK-52E that only 23% of 189 proximal-specific proteins were present in NRK-53 K cells (Khundmiri et al. 2021). In accordance, Jennings et al*.* found that human in vitro data predicted rat in vivo effects more accurately than rat in vitro data was able to in a genome wide transcriptomics study in response to the food contaminant ochratoxin A (Jennings et al. 2012). Interestingly, it was recognized that rat rPTEC may also not be a suitable model to address transport and metabolism as they have been reported to dedifferentiate rapidly when cultured in vitro. A study comparing rat gene expression levels in vivo vs. in vitro reported that approximately 80% of the enzymes involved in phase I and II biotransformation were reduced in rPTEC (Weiland et al. 2007). Moreover, most CYP-enzyme activity was drastically decreased or completely lost function within the first 24 h of culturing rPTEC. This model may be suited for investigating phase II metabolism differences since rPTEC reported enzyme activity of UGT, GST and GGT after 1 week of cell culture (Schaaf et al. 2001). Overall, it is questionable how much metabolic insight would be extrapolatable from rat in vitro systems.

## Summary and conclusions

Here, currently available human and rat kidney in vitro models, specifically renal proximal tubular cell models were reviewed, with an emphasis on expression and function of xenobiotic processing metabolizing enzymes and transporters. A particular focus was on comparison of these systems under different culture conditions, including conventional plastic culture, filter insert culture and culture in MPS. Notably, no single model currently fully represents the human situation in vivo. Nevertheless, several functional active xenobiotic metabolizing enzymes or transporters were reported in various systems. This review may therefore guide in the process of choosing a suitable human in vitro model to address the particular renal transport and/or metabolism function. Cost and throughput levels are additional important factors to consider when choosing which model to employ. A high-throughput method, and hence a good starting point for gaining more knowledge on the understanding of metabolism of a compound, is the use of renal subcellular fractions. These different fractions express the most complete range of functional renal CYP, UGT and GST enzymes. In addition, microsomes, cytosolic and S9 fractions have the benefits of being relatively inexpensive and less labor intensive. When working with microsomes, one should be aware that administration of cofactors to activate the enzymes is needed and approximately 34% to 38% of all microsomal protein is lost during the preparation of HKM (Knights et al. 2016). Testing this in a more complex system, like a cell model, offers the advantage to study phase I and phase II enzymes simultaneously. However, in human renal cell lines, functional CYP-enzyme activities have only been reported in RPTEC/TERT1 cells so far. This may not mean that other systems are not capable of functional metabolism of these enzymes, but rather that there is a lack of characterization at the functional level in other models. For example, in iPSC-derived PTL, iPSC-derived kidney organoids and kidney tubuloids there have only been reports on gene expression of phase I or phase II enzymes, but functional characterization is still ongoing. hPTEC are often expected to have the most functional phase I and phase II enzymes; however, very few studies reported on functional activity and mainly CYP3A4/5 was reportedly functionally active with large inter-individual differences in CYP metabolic capacity (Lash et al. 2008). Furthermore, different isolation techniques may make hPTEC a less predictable model as the metabolic clearance might be batch or donor dependent. One advantage of human cells systems over subcellular fractions is the inclusion of brush border enzymes that are largely absent in subcellular fractions, unless plasma membrane preparations are specifically isolated.

In order to address functional renal transport, only cell systems and no subcellular fractions are recommended. Most renal cell models, including cell lines, iPSC-derived cells and hPTEC demonstrated functional ABC efflux transport cultured under static culture condition that were not typically improved by MPS culture. Interestingly, in some cases functional ABC transport or expression levels of P-GP and MRP2/4 were reduced when cultured in MPS (Nieskens et al. 2020). In addition, functional transport of OCT varied between different cell systems. For example, HK-2 cells did not display functional OCT transport, whereas ciPTEC and RPTEC/TERT1, as well as hPTEC showed higher mRNA expression levels and reported functional OCT activity under static culture conditions (Caetano-Pinto et al. 2016; Meijer et al. 2024). Moreover, functional OCT transport was shown in iPSC-derived PTL when cultured on filter inserts (Meijer et al. 2024). In MPS systems mRNA levels of OCT transporters (OCT1, OCT2, OCTN1, OCTN2) were often comparable to statically cultured cells (Nieskens et al. 2020). It should be noted that exact alterations in expression and functionality levels varied between cell models and type of MPS used. Furthermore, not all MPS studies included static control cultures in parallel to studies performed in the MPS. Comparing data obtained in MPS with previously reported historic data from static conditions could introduce the risk that other factors, such as differences in culture conditions and cell handling, like different passage numbers and culture media, may be responsible for observed differences. In static culture, none of the immortalized cell lines reported functional OAT transport, unless they were transfected with these transporters (Nieskens et al. 2016; Sakolish et al. 2023). Some, but not all studies reported functional OAT in hPTEC (Brown et al. 2008; Bajaj et al. 2020; Sánchez-Romero et al. 2020). Culture in MPS systems on the other hand, could often improve OAT levels. For example, in hPTEC, mRNA levels of OAT were increased in MPS compared to static (Caetano-Pinto et al. 2022). Transfected cell lines seem to be an appropriate choice to evaluate whether a compound is a substrate for OATs. However, whether OAT1/3 overexpression correlates with physiological expression levels as seen in vivo is yet to be determined and the possibility that these transfected cells may be overly sensitive due to high expression, has to be investigated further.

Advanced culture conditions of cells on either static filter inserts or in MPS are highly recommended and may offer more physiologic relevant conditions with easy access to apical and basolateral sides. This may be in particular important for compounds that are taken up via transporters present on the basolateral side, that is typically less accessible when cells are cultured on plastic. In these cases, cell cultures on filter inserts, employing polarized cells that are capable of barrier formation and that express tissue-specific tight junction proteins, for example RPTEC/TERT1, iPSC-derived PTL and hPTEC, should be favored. MPS offer exciting opportunities to retrieve more stable and more mature cells; however, direct comparison studies using the same renal cell models and culture medium in MPS and static systems are underrepresented and more studies are needed to better compare potential improvement of transport and metabolism. In particular, studies on functional renal metabolism were hardly executed in MPS.

Next to improving culturing applications, like filter inserts and MPS, the cell models themselves can also be made more complex. For example, renal organoids and co-culture of renal cells with other tissue types, such as kidney and liver co-cultures, are used in studies more frequently. Functional transport in co-culture settings has not been studied elaborately, hence it is important to investigate this further. Kidney organoids have the advantage that additional renal cell types are present; however, a number of challenges still needs to be addressed, including better simultaneous access to the apical and basolateral side of the organoids to apply substrates and inhibitors for transport assays selectively. In addition, the two most commonly used protocols for iPSC-derived kidney organoids resulted in 11% and 22% non-renal cells which are primarily neurons (Wu et al. 2018). Other challenges of kidney organoids are long term stability and reproducibility. Fibrotic cells have been reported after prolonged culture (Yousef Yengej et al. 2023). Reproducibility is an issue that should be addressed, as both organoid size and the percentages of individual renal cell types may vary greatly between different differentiation batches. Additional research is required prior to recommending organoids as a more suitable model for ADME studies; however, recent advances such as improved OAT functionality make them a promising cell culture method to be studied more extensively.

## Data Availability

Not applicable.

## Abbreviations

6-CF: 6-Carboxyfluorescin
ABC: ATP-binding cassette
ADME: Absorption, distribution, metabolism, excretion
AO: Aldehyde oxidase
ASP: 4-(4-(Dimethylamino)styryl)-*N*-methylpyridinium iodide
CES: Carboxylesterase
ciPTEC: Conditionally immortalized proximal tubular epithelial cells
CYP: Cytochrome P450
EROD: Ethoxyresorufin-*O*-deethylase
GGT: γ-Glutamyl transpeptidase
GSH: Glutathione
GST: Glutathione-*S*-transferase
HK-2: Human kidney 2
HKM: Human kidney microsomes
HLM: Human liver microsomes
hPTEC: Primary human renal proximal tubular epithelial cells
hTERT: Human telomerase reverse transcriptase
iPSC: Induced pluripotent stem cell
LRP2: Low-density lipoprotein receptor-related protein
MATE: Multidrug and toxin extrusion transporter
MPS: Microphysiological systems
MRP: Multidrug resistant proteins
NA^+^/K^+^-ATPase: Sodium potassium ATPase
NAMs: New approach methodologies
NRK-52E: Normal rat kidney cells
OAT: Organic anion transporter
OATP: Organic anion-transporting polypeptides
OCT: Organic cation transporter
OCTN: Organic cation/carnitine transporter novel
OoC: Organ-on-a-chip
PAPS: 3′-Phosphoadenosine-5′-phosphosulfate
PBK: Physiological based kinetic
PDMS: Polydimethylsiloxane
P-GP: P-glycoprotein
PTL: Proximal tubular-like cells
RKM: Rat kidney microsomes
RPTEC/TERT1: Tert-transfected human renal proximal tubular epithelial cells
rPTEC: Primary rat renal proximal tubular epithelial cells
SLC: Solute carrier family
SULT: Sulfotransferase
SV40T: SV40 large T antigen
TEER: Transepithelial electrical resistance
UGT: UDP-glucuronosyltransferase
